# Academic resilience in education: the role of achievement goal orientations

**Published:** 2014-01

**Authors:** BAHRAM JOWKAR, JAVAD KOJURI, NAEIMEH KOHOULAT, ALI ASGHAR HAYAT

**Affiliations:** 1 Educational Psychology Department, Education & Psychology School, Shiraz University, Shiraz, Iran;; 2 Quality Improvement in Clinical Education Research Center, Education Development Center, Shiraz University of Medical Sciences, Shiraz, Iran;; 3 Educational Psychology Department, Kharazmi University, Tehran, Iran;

**Keywords:** Resilience, Education, Achivement

## Abstract

**Introduction**: In the past 2 decades, research findings established achievement goal theory as a powerful framework for conceptualizing difference in the quality of student's engagement, persistence on task, and academic resilience. So the present study examined the relationship between achievement goal orientations and academic resilience.

**Method**: Participants were 606 students (307 girls and 297 boys) selected from Shiraz high schools. They completed the Achievement Goals Questionnaire and Youth development Module Scale (RYDM). To analyze the data, the relationships between variables were examined, using Pearson product–moment correlations. Also simulanteous multiple regression was performed to investigate the prediction of academic resilience by achievement goal orientations. To examine the reliability and the validity of measures Cronbach alpha coefficient and factor analysis method were applied, respectively.

**Results**: Simultaneous multiple regression of achievement goal orientations on academic resilience showed that "mastery-approach" was a significant positive predictor of the "home care/high" and "peer care" subscales. Also, "performance-approach" was a significant, positive predictor of "home care/high", and "school/community meaningful" was predicted by "performance-avoidance" positively.

**Conclusion**: According to the findings, it seems that achievement goal orientation has a critical role in students’ academic achievement. Implications of the results are discussed in more details.

## Introduction

Reaserch on resilience has grown rapidly since the last decade, particularly in developmental psychology, family psychology, counseling, rehabilitation and clinical psychology (1). Resilience has been conceptualized as an individual characteristic (2) and the term resiliency has been used to refer to good, stable, and consistent adaption under challenging conditions (3).

Recently, some researchers have considered the resilience as a domain specific concept. This approach suggested different aspects of resilience, such as academic, emotional, behavioral, etc. Academic resilience took more attention between different aspects. Wang, Haertel & Walberg (1994) suggested the academic resilience as the heightened likelihood of success in school despite environment adversities brought about by early traits, conditions, and experiences (4). In other words, resilient students sustain high levels of achievement motivation and performance despite the presence of stressful events and conditions that place them at risk of doing poorly in school and ultimately dropping out of school (5). So the role of motivation may be central to educational resilience (6).

In recent years, the new wave of research in the field of mental health has focused on predictors and outcomes of academic resilience. According to current models, the factors affecting academic resilience can be catecorized as external and internal protective factors.

External protective factors are the environmental social supports and opportunities available in the home, school, community, and peer groups, in the form of a) caring relations, b) high expectations, and c) encouragement for participation in meaningful activities (6).

Internal protective factors are individual qualities and characteristics (skills, attitudes, beliefs, and values) associated with positive developmental outcomes. The internal protective factors of cooperation and communication, empathy, strong problem-solving skills, well-defined goals and aspirations, high self-efficacy, and self-awareness develop both naturally and in response to environmental protective factors, and they contribute to positive academic, social, and health outcomes (7).

The present study focused on achievement goal orientations as an important internal protective factor. Achievement goal orientations are viewed as the purpose (8, 9) or cognitive-dynamic focus of competence-relevant behavior. Broadly defined, achievement goals reflect the desire to develop, attain, or demonstrate competence at an activity (10, 11). Competence is at the conceptual core of the achievement goal construct. Competence and, therefore, achievement goals, may be differentiated on two fundamental dimensions-according to how it is defined and according to how it is valenced. Competence is defined in terms of the referent or standard that is used in performance evalution. Three different standards may be identified: absolute (the requirements of the task itself), interapersonal (one's own past attainment or maximum potential attainment), and normative (the performance of others) (12). The other fundamental dimension of competence is valence. Competence is valenced in that it is either construed in terms of a positive, desirable possibility (i.e., success) or a negative, undesirable possibility (i.e., failure) (13, 14). From orthogonal relationship between these dimensions, different types of goals will be produced including mastery-approach goals (in which competence is defined in absolute/interpersonal terms and is positively valenced), mastery-avoidance goals (in which competence is defined in absolute/interpersonal terms and is negatively valenced), performance-approach goals (in which competence is defined in normative terms and is positively valenced), and performance-avoidance goals (in which competence is defined in normative terms and is negatively valenced (11, 15).

A variety of studies has shown that different goal orientations determine students’ cognitive, affective and behavioral reactions as well as the students’ educational performance (13, 15). Academic resilience is considered as an affective consequence of goal orientations.

Generally, it is assumed that students are more satisfied and achieve better performance if they pursue a mastery orientation or a more intrinsic motivation (16). In addition, individuals with mastery goals seek out challenge and persist at task even in face of difficulty (10). In contrast, individuals with performance goals, are likely to avoid challenge and to withdraw effort or give up, especially when they are low in perceived competence (17). For example, Sideridis, and Kaplan (2011) (18) examined the role of achievement goals in students’ persistence. Results suggested that mastery-oriented students persisted significantly longer compared with performance approach–oriented, performance avoidance–oriented, and amotivated students across failure trials. However, performance approach–oriented students were more likely to rebound after experiencing success. However, some studies have failed to find such results (19-21). So they suggest that performance goals do not always have negative effects, even for individuals low in perceived competence, and mastery and performance goals can interact positively to promote adaptive behaviors (18, 22-24).

Moreover, researchers have shown that goal orientations have some deep effects on life satisfaction and well-being. For instance, goals related to self-improvement and growth (task-oriented goals) are positively associated with various indices of well-being, adjustment and life satisfaction, whereas avoidance tendencies are linked with different types of adjustment problems (25-29). Indeed, using a sample of affluent adolescents, Travers, Bohnert, & Randall (2013) investigated the mediating role of goal orientation (GO) on the relationship between school motivational climate (MC) and adolescent adjustment (30). Adolescents from four high schools completed measures of MC (i.e., Performance and Mastery), GO (i.e., Ego and Task), and adjustment (i.e., depressive symptoms, anxiety, and life satisfaction). The results showed that adolescents with higher ego orientation reported more depressive and anxiety symptoms, while adolescents with higher task orientation indicated fewer depressive symptoms and greater life satisfaction.

Also, researchers showed that, learning orientation and avoidance-oriented students were positively related to academic adjustment and academic well-being (i,e., school value, school burnout, schoolwork engagement, satisfaction with educational choice), whereas performance orientations and mastery-oriented students were not related to adjustment and academic well-being (31). In addition, academic satisfaction was positively influenced by mastery-approach and negatively influenced by performance-avoid (32). In their study, Splan, Brooks, Porr, & Broyler (2011) also examined the relation between resiliency and achievement goal orientation among agricultural students. One of their results was that resiliency and mastery-approach goal orientation were positively and moderately correlated (33). Indeed, Valencia (1994) showed that students who were goal-oriented (e.g., degree attainment) revealed greater academic persistence than those who did not possess this personal resource. So the goal-directedness appears to be the most salient predictor of academic resilience (34).

More recent studies point out that the different goal orientations do not necessarily need to be treated as opposites. Actually, multiple goals interact and jointly can influence the students positively (35). For example, Roebken (2007) suggested that students pursuing both mastery and performance goals were more satisfied with their academic experience than students who pursued a mastery orientation alone or a work-avoidance/performance orientation (36).

In this connection, the goal of present study was to examine the effects of goal orientations on academic resilience. On the basis of the theory and research summarized above, we hypothesized that:

1- Mastery-approach orientation is a positive predictor of academic resilience.

2- Performance-approach and performance-avoidance orientations will negatively predict academic resilience.

## Methods

The research method was descriptive-correlative. The participants were 626 high school students (316 girls and 310 boys) that were selected by multi-stages cluster random sampling from different high schools of Shiraz. They completed a self-report questionnaire tapping goal orientations and academic resilience in the classroom during a 45-min school lesson. The participants whose questionnaires were completed incorrectly were excluded from the final analyses (N=20). Therefore, in the final analysis there were 606 students from high schools. All descriptive statistics, regression and confirmery factor analyses were performed with SPSS 14 (SPSS Inc, Chicago IL, USA). To analyze the data, relationships between variables were examined, using Pearson product–moment correlations. Also simulanteous multiple regression was performed to investigate the prediction of academic resilience through achievement goal orientations.

**Measures**

**Achievement Goals Questionnaire (AGQ):** The students’ achievement goal orientation was measured, using Elliot and McGregor's (2001) achievement goal questionnaire, which comprises four subscales of mastery-approach, mastery-avoidance, performance-approach, and performance-avoidance (14). The AGQ is a twelve item scale (3 items were devised for each goal) which allows responses ranging from 1 (not at all true of me) to 7 (very true of me). Sample items are: “I desire to completely master the material presented in this class” (mastery-approach), “I worry that I may not learn all that I possibly could in this class” (mastery-avoidance), “It is important for me to do better than other students” (performance-approach), and “My goal in this class is to avoid performing poorly” (performance-avoidance).

A preliminary study of the psychometric properties of the scale showed that it had adequate reliability and validity (Elliot & McGregor, 2001). However, in the present study, to determine the reliability of the scale, Cronbach alpha coefficient was calculated. Alpha coefficient for mastery-approach was 0.87, for mastery-avoidance 0.89, for performance-approach 0.84 and for performance-avoidance 0.53. Also, the principal components analysis (PCA) was used to examine the validity of the scale. The factor analysis confirmed 4 factors and showed that all items were highly loaded on one factor.

**Resilience and Youth Development Module (RYDM)**

The RYDM used in this study was the M6 2002 version of the Middle School Resilience and Youth Development Module, which is an optional module of the California Healthy Kids Survey (CHKS). It was developed for the California Department of Education (CDE), a non-profit research, development, and service agency. This scale is intended for use in assessing and understanding a variety of external and internal protective factors associated with positive youth development (7). Specifically, the external assets subscales of the scale ask students their perceptions of caring relationships, high expectations, and opportunities for meaningful participation in their home, school, community, and peer group. The internal assets subscales of the scale measure personal strengths associated with healthy and successful development including cooperation and communication, empathy, problem solving, self-efficacy, self-awareness, and goals and aspirations. The instrunment has a 4-point Likert scale, ranging from “very much true = 4”, “pretty much true =3”, “a little true =2”, to “not at all true = 1”. The Students are instructed to indicate the degree to which each item in the module applies to them.

In this study, five subscales of external factors were examined. These external factors were "community care/high", "school care/high", "home care/high", "peer care" and "school community meaningful". A preliminary study of the psychometric properties of the scale showed that it had adequate reliability and validity. However, in the present study, to determine reliability of the scale, Cronbach’s alpha coefficient was calculated. Alpha coefficient for “community care/ high” was 0.88 for “school care/ high” 0.80, for “home care/ high” 0.83, for “peer care” 0.79, and for “school/ community meaningful” 0.78. Also, Principal components analysis (PCA) was used to examine validity of the scale. The factor analysis confirmed 5 factors and showed that all items were highly loaded on one factor.

## Results

**Descriptive statistics**

Using SPSS 14, descriptive statistics such as mean and standard deviation for all of the variables used in the study were examined (Table 1).

**Table 1:** Descriptive statistic of variables

**Variables**	**Mean±SD**
**Mastery-approach**	12.704±2.553
**Mastery-avoidance**	10.411±3.054
**Performance-approach**	12.106±2.723
**Performance-avoidance**	9.464±2.928
**Community care/high**	17.635±5.288
**School care/high**	18.254±4.685
**Home care/high**	25.134±5.0732
**Peer care**	13.598±3.700
**School/community meaningful**	18.887±5.455

**Correlations**

For analysing the data, at first correlations between the measured variables were calculated. The Pearson correlations between all the measures are shown in Table 2. As is shown, the results revealed that there were a positive and significant correlations between mastery-approach with home care/high and peer care, performance-approach with home care/high, and performance-avoidance with school/community meaningful.

**Table 2:** Correlation matrix of achievement goals and academic resilience

**Variable**	**1**	**2**	**3**	**4**	**5**	**6**	**7**	**8**	**9**
**1. Community care/ high**	1								
**2. School care/ high**	0.203**	1							
**3. Home care/ high**	0.353**	0.254**	1						
**4. Peer care**	0.251**	0.284**	0.333**	1					
**5. School/ community meaningful**	0.304**	0.381**	0.304**	0.331**	1				
**6. Mastery-approach**	0.022	0.061	0.251**	0.114*	0.012	1			
**7. Mastery-avoidance**	-0.072	-0.043	0.023	-0.032	0.041	0.324**	1		
**8. Performance-approach**	0.063	0.062	0.201**	0.081	0.041	0.583**	0.361**	1	
**9. Performance-avoidance**	-0.044	-0.051	-0.054	-0.044	0.101*	0.162**	0.481**	0.363**	1

* p<0.05                ** p<0.001

**Regression analysis**

Simulanteous multiple regression was performed to investigate the prediction of academic resilience by achievement goal orientations. The results showed that "mastery-approach" and "performance-approach" was a significant positive predictor of the "home care/high". In addition, performance-avoidance positively predicted school/community meaningful and mastery-approach was a significant positive predictor of the peer care. The results are summarized in Tables 3 and 4.

**Table 3:** Multiple regression of achievement goal orientations on academic resilience

**Variables**	**Community care/ high**				**School care/ high**				**Home care/ high**			
	** R^ 2 ^**	**B**	**β**	**p<**	** R^ 2 ^**	**B**	**β**	**p<**	** R^ 2 ^**	**B**	**β**	**p<**
**Constant**	0.012	17.633		0.000	0.02	16.715		0.000	0.084	18/911		0.000
**Mastery-approach**		0.006	0.003	n.s		0.146	0.080	n.s		0.453	0.219	0.000
**Mastery-avoidance**		-0.159	-0.093	n.s		-0.075	-0.050	n.s		-0.070	-0.042	n.s
**Performance-approach**		0.176	0.089	n.s		0.103	0.061	n.s		0.223	0.118	0.032
**Performance-aviadance**		0.056	-0.031	n.s		-0.091	-0.058	n.s		-0.169	-0.098	n.s

**Table 4:** Multiple regression of achievement goal orientations on academic resilience

**Variables**	**Peer care**				**School/ community meaningful**			
	** R^ 2 ^**	**B**	**β**	**p<**	** R^ 2 ^**	**B**	**β**	**p<**
**Constant**	0.023	11.735		0.000	0.013	16.539		0.000
**Mastery-approach**		0.172	0.119	0.031		0.006	0.003	n.s
**Mastery-avoidance**		-0.074	-0.063	n.s		0.007	0.004	n.s
**Performance-approach**		0.082	0.061	n.s		0.003	0.001	n.s
**Performance-aviadance**		-0.050	-0.041	n.s		0.205	0.111	0.035

Also the results of t-test analysis showed there were no significant differences between girls and boys in all variables. So the effect of sex variable was omitted in other examinations.

## Discussion

The present article sought to examine the effects of goal orientations in predicting academic resilience in the high school setting. Overall, the results from this study contribute to the literature by providing empirical evidence that four factor models of goal orientation has utility in predicting dimensions of academic resilience.

In the line with previous research (10, 16, 27, 29), mastery-approach goal orientation was a positive predictor of academic resilience. Especially, individuals high in mastery-approach had higher levels of "home care/high" and "peer care" dimensions. This may be due to the fact that for mastery-oriented students, an important goal is to learn and understand as much as possible, yet they also stressed the importance of getting goal grades (29). Moreover, for them, engagement in a task arises from an inherent need for growth, learning, and improvement. So, even in the case of failure or another problematic situation, mastery-oriented individuals appraise the situation as an opportunity to learn and grow from their mistakes (24). Thus, these students have high level of academic resilience in the face of difficulty.

Perhaps the most interesting findings in this study exhibited that performance goals (approach & avoid) was a significant, positive predictor of academic resilience. Actually, the positive impact of performance goals is consistent with many prior research showing that individuals with performance goals have different types of adjustment problems (17, 26, 27, 29, 37). However, some research showed these goals can influence students positively (22-24, 36). As Harackiewicz, Barron, and Elliot (1998) (38) and Dykman (1998) (39) have suggested, the intrinsic value associated with the adoption of competence-based goals may account for enhanced effort and persistence. Recent empirical findings corroborate the idea that under specific circumstances (e.g. when perceived competence is not devalued), performance-approach goals, may be adaptive and, at times, even more adaptive campared with mastery goals (see Harackiewicz, Barron, Carter, Lehto, & Elliot, 1997; Midgley et al., 2001) (21, 40).

As shown by the results, contrary to the existing literature, goal orientation wasn’t a predictor of academic resilience. Population disparity and the sampled group might be the causes. Since Iranian education system focuses more on comparing the students’ performance with one another and/or the predetermined criteria (e.g. grades), the students have developed a performance-avoidance orientation (on the grounds of counter-posing themselves with others, and that fear of grades and exams has turned out to be an important reason for the Iranian students to study), and therefore, goal orientation couldn’t, in the other aspects, predict academic resilience on account of the fact that the sample was homogenous in terms of this characteristic as well as lack of variance in the variable under study. 

There are some limitations of this study. It is important to note that the generalizability of our results may be limited to high school student population. Based on the literature, these goal orientation findings should also be generalized to other populations such as younger school-age students. 

Several theoretical implications results from this study. First, other variables that may explain students’ academic resilience should be examined since they may have incremental explanatory power goal orientations. Second, managers, coaches, and teachers may use these results to help individuals develop more adaptive goal orientations.

## Conclusion

Finally, there is a question that needs to be answered in the future. It is important to evaluate the antecedents of goal orientations, which could provide explanations of why performance goals sometimes prove to be adaptive and sometimes not. Also based on the results, we recommend replicating the relations in other groups. In case the results of the replications are the same, the characteristics of the groups should be taken into account to figure out the root cause(s) so that we could change the education system for good, and on the other hand shift the focus away from grades and performance to mastery and skill development.
